# Advances in biomarkers and treatment strategies for HPV-associated head and neck cancer

**DOI:** 10.18632/oncoscience.425

**Published:** 2018-06-25

**Authors:** Cassie Pan, Wendell G. Yarbrough, Natalia Issaeva

**Affiliations:** Department of Surgery, Division of Otolaryngology, Yale Cancer Center, Yale University, New Haven, CT, USA

**Keywords:** head and neck cancer, HPV, 5-azacytidine, TRAF3, CYLD

Understanding the etiology of human papillomavirus (HPV)-driven oropharyngeal squamous cell carcinoma (OPSCC) is becoming a matter of increasing necessity and urgency as the incidence of HPV(+) OPSCC continues to rise and now exceeds that of HPV-associated cervical cancers [1]. HPV(+) head and neck cancers, over 90% of which are caused by HPV type 16, are clinically and epidemiologically distinct from those not associated with HPV. Moreover, HPV(+) and HPV(−) OPSCCs display distinct mutation and methylation landscapes and different protein expression profiles [2, 3]. Regardless of treatment strategy, HPV-positivity confers a favorable prognosis with 5-year survival rates of 75-80% versus 45-50% among HPV-negative patients [4]. Despite these differences, treatment guidelines recommend similar treatment regardless of HPV status. Efforts to de-escalate therapy for HPV(+) patients are now not only being prioritized to maintain cure rates, but also to decrease treatment-related side effects.

Treatment for advanced head and neck cancer includes radiation and platinum-based chemotherapy, which are associated with dose-related adverse side effects including acute toxicities like mucositis and loss of taste, as well as long-term problems of dysphagia, osteoradionecrosis, xerostomia, muscle fibrosis, and lymphedema. These side effects can lead to downstream infections, difficulty eating, and increased hospitalizations, which decrease a patient's quality of life. Limiting these side effects is especially important in HPV(+) patients, who present at a younger age. Despite their improved cure rates, 20-30% of HPV(+) tumors will recur and upon recurrence, effective treatment options are limited.

Given toxicities associated with aggressive therapy, recent studies have focused to improve treatment of HPV(+) OPSCC. The most common strategies include limiting platinum-based chemotherapies and decreasing radiation fields or dosage. At best, these strategies can minimize therapeutic side effects, but at the risk of decreased cure rates. Our group has taken a different approach with two major points of focus. The first has been to identify vulnerabilities of HPV-associated OPSCC that may lead to more targeted toward HPV(+) tumors treatments. The second has been to identify reliable biomarkers that predict which patients can benefit from and safely undergo treatment de-escalation. We hope that these research directions will not only decrease side effects while maintaining efficacy, but also provide treatment for those with recurrent or resistant HPV(+) OPSCC.

**Figure 1:**
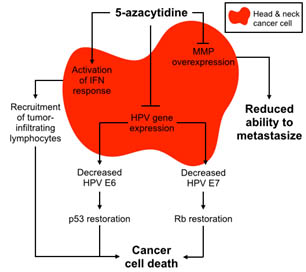
Schematic of selective multimodal 5-aza toxicity in HPV+ HNSCC

Recently, we became interested in 5-azacytidine (5-azaC), a DNA-demethylating agent, following the discovery that HPV(+) tumors have a hypermethylated genome [3]. Previous literature has shown that demethylation decreased expression of the major HPV oncogenes *E6* and *E7*, which have been linked to hypermethylation and invasiveness in cervical cancer [5, 6]. In our preclinical studies and in a window clinical trial at the Yale Cancer Center, 5-azaC markedly downregulated expression of all HPV genes. 5-azaC treatment of cultured cells inhibited growth and caused cell death that was dependent on stabilization of p53 (Figure 1) [7]. Increased p53 protein levels following 5-azaC correlated with decreased expression of the HPV E6, providing a mechanism for this effect. We also examined the ability of 5-azaC to inhibit tumor metastasis. Using cell culture, xenograft mouse model, and samples from the window clinical trial, we found that 5-azaC decreased expression of matrix metalloproteinases (*MMPs*) *1* and *10* that degrade the extracellular matrix to allow cancer cells to spread and have been shown to be critical for carcinomas invasion. Consistent with downregulation of MMPs, we found that xenografted HPV(+) OPSCC had reduced metastatic potential after 5-azaC treatment [7].

The ability of 5-azaC to activate interferon (IFN) response and induce a local production of T cells attracting cytokines in some HPV(+) head and neck cancer cells was another important finding of our study, suggesting a therapeutic potential of a combination of 5-azaC with anti PDL1/PD1 immunotherapy. Together, our data proposes the potential of 5-azaC in the treatment of HPV(+) OPSCC and prevention of metastasis (Figure 1) and warrants investigation in a larger clinical trial.

One challenge of therapeutic de-escalation is to determine the appropriate subset of patients that will not suffer worse prognosis as a result. To address this challenge, we sought biomarkers that have utility in selecting HPV(+) OPSCC patients with improved prognosis who may be candidates for de-escalated therapy. Our analysis of The Cancer Genome Atlas (TCGA) head and neck cancer database revealed nearly mutually exclusive inactivating mutations in TRAF3 and CYLD genes in 28% of HPV(+) specimens [8]. TRAF3 and CYLD serve functions in activating immune response and inhibiting NF-kB, which is known to be activated in many cancers. These gene defects were also found to occur in 25% of a separate cohort of 23 HPV(+) OPSCC patients treated at Yale (data not shown), confirming that a subset of HPV(+) OPSCCs with defective TRAF3/CYLD may rely on overactive NF-kB and impaired innate immunity. Remarkably, analysis of the TCGA cohort revealed improved survival in HPV(+) patients with TRAF3/CYLD mutations compared with wild-type TRAF3/CYLD, while survival of HPV(+) patients without these mutations was similar to that of HPV(−) negative patients. These results suggest that constitutive activation of NF-kB defines a subgroup of HPV(+) patients with improved survival for whom de-escalated therapy may be safe and effective.

Development of less toxic treatment options and identification of a patient population that may be best for testing these therapies are key to improving patient morbidity and mortality in HPV(+) OPSCC. As more clinicians and researchers recognize this distinct subgroup of tumors and more is discovered about HPV-driven carcinogenesis, the better equipped we will be to prevent and treat HPV(+) head and neck cancer patients.
